# Efficacy of Panoramic Radiography in the Detection of Osteoporosis in Post-Menopausal Women When Compared to Dual Energy X-Ray Absorptiometry

**DOI:** 10.2174/1874210601711010350

**Published:** 2017-06-30

**Authors:** Shambulingappa Pallagatti, Priyanka Parnami, Soheyl Sheikh, Deepak Gupta

**Affiliations:** 1 Department of Oral Medicine and Radiology, M.M. College of Dental Sciences and Research, Mullana, Ambala, Haryana, India; 2 Department of Oral Medicine and Radiology, Pacific College of Dental Sciences and Research, Udaipur, Rajasthan, India

**Keywords:** Bone mineral density, Dual energy X-ray Absorptimetry, Klemetti index, Panoramic radiographs, Post-menopausal women, Osteoporosis, Mandibular cortex

## Abstract

**Objective:**

It is a well-known fact that osteoporosis affects the people with old age and remains unnoticeable until the patient presents with fracture. Various studies in the literature reveal that panoramic radiographs may prove to be beneficial in the detection of Osteoporosis in patients. Henceforth this present study was aimed to validate the use of Klemetti Index (KI) on panoramic radiographs so as to detect osteoporosis in the patients at an early stage.

**Methods:**

60 postmenopausal women were selected. A panoramic radiograph was taken to grade their mandibular cortex on the basis of Klemetti Index. All the panoramic radiographs were evaluated by 5 different Oral Medicine and Radiology specialists. Later all the patients were subjected to dual energy X-ray absorptimetry (DEXA) scan for bone mineral density evaluation. The results were evaluated statistically.

**Results:**

The average accuracy of the five observers to demonstrate normal bone, osteopenia and osteoporosis when compared to DEXA scan was 58.08%, 63.3% and 64.74% respectively. The observations of the 5 observers on the basis of KI were not statistically different from the BMD evaluation done with the help of DEXA Scan.

**Conclusion:**

Panoramic radiographs can be used as a screening tool for the evaluation as well as early detection of osteoporosis with the usage of Klemetti Index.

## INTRODUCTION

Osteoporosis is one of the most prevalent metabolic bone diseases and is manifested by the loss of bone mass which further presents with micro architectural deterioration in bone tissues [1-16]. This in turn leads to structural changes in the bone [1-16]. It affects mainly the people with old age and might not be detected unless the symptoms of fracture occur [1-3, 16].Such osteoporotic fractures occur due to trauma to weak bones of the skeleton. These fractures commonly occur at the sites composed predominantly of medullary bone [2,17]. It is a well known fact that this disease is preventable. Henceforth, the diagnostic techniques which can help in the early detection of the disease are of major importance [18].

Nowadays, with the growing number of elderly people, the incidence of osteoporosis has also increased [3,19-23]. It is widely recognized as a major public health concern [4] and is estimated to affect up to one third of post menopausal women [5]. Osteoporosis can lead to many types of fractures, but the hip, spine, forearm and shoulder are the most common sites [3, 6, 16]. Literature also reveals that even Hormonal disturbances can also play a major role in the reduction of bone mineral density [7, 18]. There is an evidence that genetic variants in various hormones, cytokines and their receptors that are involved in bone remodeling may also contribute to the development of osteoporosis [8, 9].

Literature reveals that the status of bone at various sites may be assessed with the help of dual energy X-Ray absorptiometry (DEXA), quantitative ultrasound (QUS) or quantitative computed tomography (QCT) [10, 15]. Bone mineral density (BMD) assessment by DEXA (Dual energy x-ray absorptiometry) is considered the gold standard for identifying osteoporosis [11]. It is a good diagnostic technique because it has good accuracy and precision in detecting low bone mass [12].

Several studies have shown that large segments of the population of postmenopausal women with an age of 50 years and above with no previous diagnosis of osteoporosis may present with low skeletal bone mineral density (BMD) [21]. Henceforth, BMD testing for postmenopausal women may be beneficial to reduce the incidence of osteoporotic fractures and subsequent complications [21-26]. On the contrary it is also obvious that such mass screening of bone mineral density by DEXA is not feasible. Further, it is important to note that literature provides good evidence regarding the significant relation of mandibular bone mineral content to generalized skeletal osteoporosis. Therefore, dental radiography may prove helpful as a simple means of screening patients for osteoporosis [13].

The earliest suggestion of an association between osteoporosis and oral bone loss was made in 1960 [18]. Recent studies have also shown that there are changes in the cortical layers of the mandible, which in turn depend upon the age and mineral loss within the skeleton [27-29]. These changes can easily be seen on maxillofacial panoramic radiographs. Further it is of interest to note that performing a panoramic radiograph for dental and maxillofacial diseases is a frequent task [21-26]. Henceforth there are good chances that the incidental cortical erosion of mandible if detected on the panoramic radiograph may help identify women susceptible to osteoporosis who may not be aware of their low bone mineral density [17-19]. Therefore, dentists are in a potentially advantageous position for patient screening regarding signs of osteoporosis [17] and may be the first amongst the health professionals who may spot an undiagnosed patient with osteoporosis or the patients who are at risk to develop osteoporosis at a later stage.

Hence, the aim of this study was to determine the validity of the Klemetti Index (KI) measured on panoramic radiographs, in the diagnosis of osteoporosis so as to ascertain the possibility of prediction of skeletal BMD from the changes on the panoramic radiographs in post-menopausal women.

### MATERIALS AND METHODS

60 post menopausal women of Indian origin aged more than 45 years were selected for the study. The subjects were randomly selected from the out patients visiting the department of Oral Medicine and Radiology. Time period for the study was in between the year 2010-2011. All subjects were advised panoramic radiographs for dental purposes. Further, they were explained the need and design of the study and the benefit of undergoing through clinical, radiographic and BMD investigations. Subjects who agreed to undergo these procedures were included in the study and instructed to read and sign the consent form. The postmenopausal women who had a history of chewing or smoking tobacco, betel quid with slaked lime or medications that affect bone metabolism were excluded from the study. Even the subjects from the regions of Endemic fluorosis were excluded as the fluoride can alter bone density. The subjects who have undergone hysterectomy or oophrectomy or who had metabolic bone diseases, diabetes, significant renal impairment, bone-destructive lesions in the jaw and non-vertebral osteoporotic fractures were also excluded. Institutional ethical clearance was obtained.

The subjects were made to sit comfortably on the physiological dental chair with artificial illumination and routine dental check up was done. The data such as name, age, sex, address, occupation, chief complaint or any significant medical history, dental history and duration of menopause was recorded in the proforma. All panoramic radiographs were obtained with an Advapex digital panoramic machine at 12 mA and 14 seconds with 75 kVp. Print outs of the radiographs were then obtained on a radiographic film using Konika printer (Dry Pro Model 793). All the panoramic radiographs (OPG) were evaluated by 5 different oral radiology specialists. Only print outs of the digital OPGs were provided to all the five observers so as to prevent biased interpretation.

**Fig. (1):**
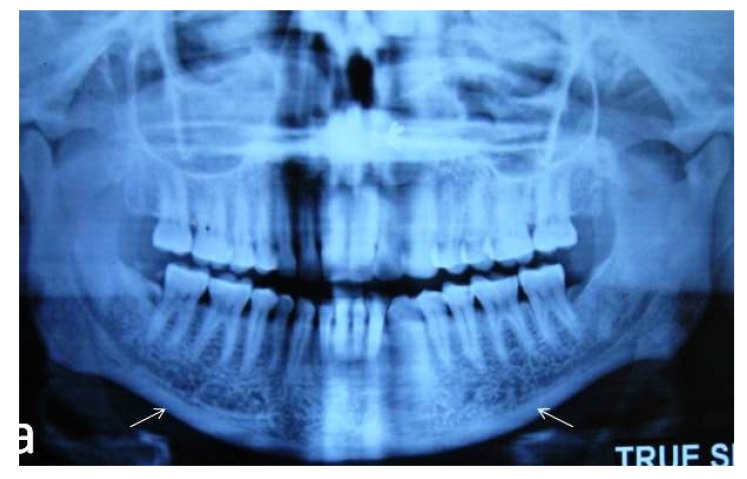
Panoramic radiograph revealing normal cortex.

**Fig. (2):**
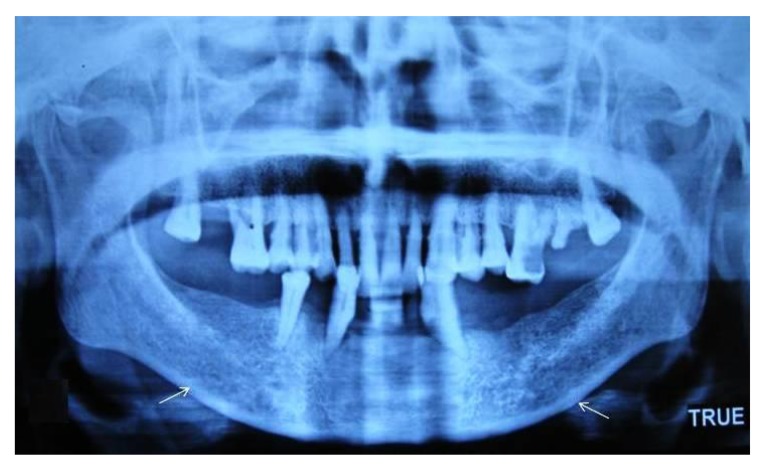
Panoramic radiograph revealing mild to moderately eroded cortex.

**Fig. (3):**
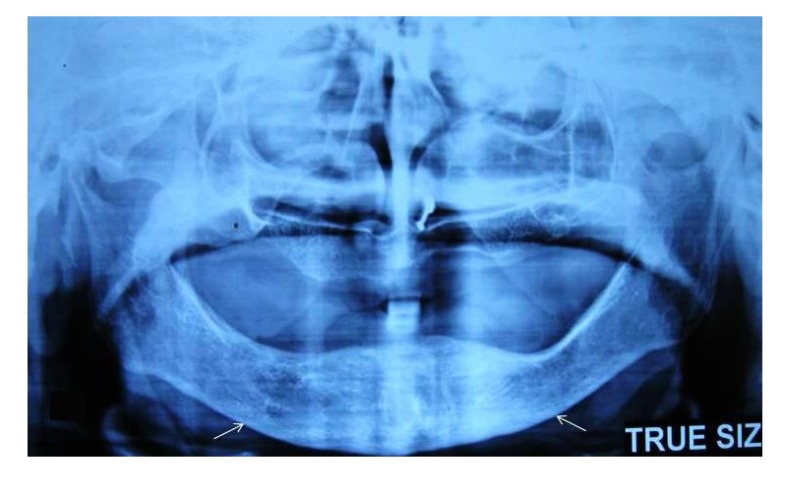
Panoramic radiograph revealing severely eroded cortex.

The thinning of inferior mandibular cortex was observed in the premolar molar region and findings of panoramic radiographs were then categorized and graded according to Klemetti Index [14-21] as follows:

- **
Normal Cortex:** The endosteal margin of the cortex is even and sharp on both sides. **(Grade 0) **(Fig.** 1**).
- **
Mildly to moderately eroded cortex:** The endosteal margin shows semilunar defects (lacunar resorption) or appears to form endosteal cortical residues **(Grade I) **(Fig. ** 2**).
- **
Severely eroded cortex:** The cortical layer forms heavy endosteal cortical residues and is clearly porous. **(Grade II)** (Fig. ** 3**).

The same group of participants was then subjected to bone mineral density tests using DEXA machine to detect the risk of osteoporosis. BMD at the lumbar spine (L1–L4 and parts of L5 and T12) was measured by a dual-energy X-ray absorptiometry (DEXA) scanner (GE Dual energy X-ray absorptiometry Machine). The in vivo short-term precision error for spine BMD of the machine used was 1.0%. The data obtained was analyzed by the software provided by the manufacturer. This data was displayed as a graph with a summary of the bone density measurements. Spine BMD was categorized as normal (T score >–1.0), osteopenic (T score,–1.0 to –2.5), or osteoporotic (T score <–2.5), according to the World Health Organization (WHO) classification [14-21]. Height and weight were measured at the time of the DEXA scanning.

The results of grading of the panoramic radiographs interpreted by the five oral radiologists and the results of the BMD tests were tabulated. These results were statistically analyzed using Statistical Package for Social Sciences (SPSS Inc., Chicago, IL, version 15.0 for Windows). Unpaired t-test was applied for the comparison of two groups. Further, Qualitative or categorical variables were described as frequencies and proportions. Chi square or Fisher’s exact test was used for comparing the proportions.

## RESULTS

All the patients were in the age range of 47-85 years with a mean age of 57.9±7.68 years. The mean height and weight were found to be 155.27±5.49 cm and 60.03±12.67 Kg respectively. All the patients on an average were found to have attained menopause at the age of 47.2 years. The mean body mass index was found to be 24.85±4.79 in all the patients.

The findings of all the observers are shown in Table **1**.There was no statistically significant intra observer difference found with *P* value of 0.186.

According to DEXA scan, out of 60 patients, 35% were found with normal bone mineral density whereas 30% were found to be osteopenic. 35% showed osteoporotic changes (Table** 2**).

The mean values of age, height, weight, age of attaining menopause and body mass index of the patients who were found to be normal and osteoporotic on DEXA scan (Table **3**) and were analyzed statistically. According to DEXA scan, the mean ages of patients presenting as Normal and Osteoporotic were 56.7 and 61.05 years respectively with no significant statistical difference between them. Similarly the mean heights of the patients presenting as normal and osteoporotic were 154.29±5.29 mm and 154.05±5.59 mm respectively on DEXA scan. This difference was also non-significant. Both the groups presented with the same mean age of occurrence of menopause at 47.0 years in normal patients and 47.86 years in osteoporotic patients with no statistically significant difference (Table **3**).

On the contrary, the difference in the weight and Body mass index was found to be statistically significant in both the groups with *P* value of 0.012 and 0.007 respectively.

There was no statistically significant difference found when the DEXA results were compared with the results of all the 5 observers individually.

As compared to the results of DEXA, Observer I correctly identified 13 normal cases, 11 cases of osteopenia and 16 cases of osteoporosis with percentage within DEXA results of 61.9%, 61.1% and 76.2% respectively (Table **4**). The accuracy for observer I was calculated according to the formula:

(correct Normal + correct Osteopenia + correct Osteoporosis) /60 x 100

*i.e*. (13+11+16)/60 x 100 = 66.67%

Similarly, Observer II correctly identified 10 normal cases out of the 15, 11 cases of osteopenia out of the 25 and 15 cases of osteoporosis out of the 20 when compared to DEXA scan with the percentage within DEXA of 47.6%, 61.1% and 71.4% respectively. Accuracy for observer II was 60%.

Observer III correctly identified 16 normal cases out of the 25, 9 cases of osteopenia out of the 20 and 10 cases of osteoporosis out of the 15 when compared to DEXA scan with percentage within DEXA of 76.2%, 50% and 47.6% respectively. The accuracy for observer III was 58.33%.

Observer IV correctly identified 10 normal cases out of the 14, 14 cases of osteopenia out of the 31 and 12 cases of osteoporosis out of the 15 when compared to DEXA scan with the percentage within DEXA of 47.6%, 77.8% and 57.1% respectively. The accuracy for observer IV was 60%.

Observer V correctly identified 12 normal cases out of the 19, 12 cases of osteopenia out of the 22 and 15 cases of osteoporosis out of the 19 when compared to DEXA scan with percentage within DEXA of 57.1%, 66.7% and 71.4% respectively. The accuracy for observer V was 65%.

The average accuracy of the five observers to demonstrate normal bone, osteopenia and osteoporosis when compared to DEXA scan was 58.08%, 63.34% and 64.74% respectively (Table ** 5**). The overall panoramic accuracy for five observers in accordance to DEXA scan was 62%.

## DISCUSSION

It is a duly acknowledged fact that early detection of osteoporosis is of utmost importance as it will lead to institution of preventive measures as well as early treatment [20, 21]. Since Osteoporosis is associated with low trauma fractures, it may lead to heavy outcomes as for as the morbidity, mortality and cost of health care is concerned [17]. Several studies have been performed regarding the efficacy of various indexes for osteoporosis [2] and to clarify whether dentists could be beneficial enough to warn for the risk of osteoporosis [2-10].

Many risk factors have been implicated for osteoporosis. They include post menopausal women, low body weight, smoking habits and use of oral glucocorticoid therapy for more than three months [17]. It further includes excessive thyroxine doses, low calcium intake, low physical activity and alcohol intake more than two times a day [2-8]. Systemic conditions associated with increased risk for osteoporosis include chronic obstructive pulmonary disease, gastrectomy, hyperparathyroidism, hypogonadism, multiple myeloma and celiac disease [2-12].

Only post-menopausal women aged 45-years and above were included in this study. This is so because according to the National Osteoporosis Foundation, bone deterioration is greater in women as compared to men and furthermore, there is increased bone deterioration in post-menopausal women [2, 16]. This may be attributed to the fact that after the menopause, the general skeletal bone mass decreases owing to estrogen deficiency, and this process often leads to osteoporosis [2].

It is a known fact that post-menopausal women constitute more than 15% of the population in developed countries [16, 27-30]. Literature also reveals that by the year 2030, the world population of menopausal and post menopausal women with osteoporosis is expected to increase to 1.2 billion [16, 27-30]. Access to screen for osteoporosis is often limited. Various methods as already described have been used to assess bone density. All these techniques use ionizing radiations to assess bone mineral density. The DEXA scan was selected because of the fact that it is considered to be very precise and accurate with negligible radiation. Moreover, it is a reproducible predictor for osteoporotic fracture and is excellent measure for response to treatment.

 DEXA can also distinguish regional as well as whole body parameters of body composition. On the contrary, the equipment of DEXA is expensive and osteoarthritis may interfere with the Spine DEXA Scan reading [16]. False elevated BMD on PA Spine views in elderly often requires trained radiology personnel to operate. However, panoramic radiographs are frequently taken in dental office for maxillofacial region and are cost-effective [2].

As far as the maxillofacial area is concerned, it has been reported that the Bone Mineral Density of mandibular cancellous bone at the level of mental foramen is related to the bone mineral density of Lumber spine in post menopausal women [14]. Furthermore, BMD of mandibular cancellous bone in osteoporotic group was found to be significantly lower than in normal group.

In the present study, Klemette index was applied on the panoramic radiographs [14, 15]. This mandibular cortical Index is a simple three-graded classification of changes in the bone cortex, which was able to distinguish normal and osteopenic/osteoporotic women [14-21]. This index was chosen because of the fact that the reproducibility of the index was found to be 98% by Klemette *et al.* [14]. Furthermore, a significant correlation was observed between the Klemette classification and vertebral BMD. To add on, in the same study, the intra and inter observer agreement was found to be substantial.

In this study, according to DEXA scan, out of 60 patients, 35% were found with normal bone mineral density, 30% were found to be osteopenic while 35% showed osteoporotic changes. This showed that bone mineral density significantly decreases in post-menopausal women.

There was no statistically significant difference found between the mean age, height as well as the age of attaining menopause in the women who presented as Normal and Osteoporotic on the basis of DEXA scan. But it was interesting to note that there existed a significant difference regarding the mean weight as well as mean BMI in between those postmenopausal women which were designated as normal and osteoporotic by DEXA scan. Hence, it can be commented that the postmenopausal women can fall prey to osteoporosis regardless of the age, height and time of menopause.

Further, each panoramic radiograph was then evaluated by 5 different Oral Radiology specialists so as to evaluate the reliability of panoramic radiographs regarding assessment of osteoporotic risk based on Klemetti Index. It was found that there was no statistically significant difference between the results of all 5 observers individually when compared to DEXA scans.

 The average accuracy of the five observers to demonstrate normal bone, osteopenia and osteoporosis when compared to DEXA scan was 58.08%, 63.3% and 64.74% respectively. The results obtained suggested that panoramic radiographs can serve as a significant adjunct in determining the osteopenic and osteoporotic changes in post-menopausal women. This was in accordance with the results obtained by Akira Taguchi *et al*. in 2007 [15].

Similarly, Anders Hailing *et al*. in 2005 concluded that the assessment of mandibular cortex patterns in panoramic radiographs according to Klemetti Index as followed in this study, was a reliable method to detect osteopenia and osteoporosis and demonstrated 87% of the correctly classified subjects [19].

Various other authors like Horner and Devlin in 1991 also confirmed the validity of using dental panoramic tomogram for the measurement of mandibular bone mineral content for the diagnosis of osteoporosis [20]. Stuart White *et al.* in 2004 stated that the dentists have sufficient clinical and radiographic information regarding the patient so as to play a good role in screening for individuals with osteoporosis [21].

So, it was concluded that the dentists can refer postmenopausal women for bone densitometry on the basis of incidental findings on dental panoramic radiographs [15]. This study has been supported by Akira Taguchi *et al* in 2003 and several others who suggested that the findings on dental panoramic radiographs may be an indicator of bone turnover as well as spine BMD in postmenopausal women [22-26].

 On the contrary some of the authors were of the opinion that dental panoramic radiographs don’t provide adequate information for the diagnosis of osteoporosis [21-26]. Further literature even provides the reference of the possibility to show positive correlations between BMD of skeleton and changes in the mandibular cortex on the panoramic images [27-29] when large sample size was used. This same point has been confirmed in this study. Some of the authors commented that the osteoporosis risk for even a single person can be diagnosed with certainty by using panoramic X-ray images [26].

Henceforth, in agreement with several studies [31, 32], it can be concluded that panoramic X-ray images can yield suitable information to diagnose patients at the risk of osteoporosis. Furthermore, in the light of panoramic radiographs being highly prescribed with added advantageous features of low cost as well as ease of interpretation, they can be widely used to assess asymptomatic population regarding osteoporosis risk. Hence, the findings of panoramic radiographs can serve as a useful method for osteoporosis detection and referring the patients for further investigations [33, 34].

## Figures and Tables

**Table 1:** Panoramic radiographic observation by 5 observers.

	**Observer I**	**Observer II**	**Observer III**	**Observer IV**	**Observer V**
**Normal**	17 (28.3)	15 (25.0)	25 (41.7)	14 (23.3)	19 (31.7)
**Osteopenia**	19 (31.7)	25 (41.7)	20 (31.3)	31 (51.7)	22(36.7)
**Osteoporosis**	24 (40.0)	20 (33.7)	15 (25.0)	13 (21.7)	19 (31.7)
**Total**	60 (100.0)	60 (100.0)	60 (100.0)	60 (100.0)	60 100.0)

**Table 2:** Bone mineral density tests according to DEXA scan.

	**Frequency**	**Percent**
Normal	**21**	35.0
Osteopenia	**18**	30.0
Osteoporosis	**21**	35.0
Total	**60**	100.0

**Table 3:** Comparison of normal and osteoporosis group by DEXA scan according to body mass index.

Group Statistics					
	**Dexa Results**	**N**	**Mean**	**Std. Deviation**	**Std. Error Mean**
**Age (yrs)**	**Normal**	21	56.71	9.429	2.058
	**Osteoporosis**	21	61.05	6.265	1.367
**Ht. (cm)**	**Normal**	21	154.29	5.293	1.155
	**Osteoporosis**	21	154.05	5.590	1.220
**Wt. (kg)**	**Normal**	21	65.52	14.511	3.167
	**Osteoporosis**	21	54.71	11.845	2.585
**Age at Menop. (yrs)**	**Normal**	21	47.00	4.087	.892
	**Osteoporosis**	21	47.86	2.670	.583
**BMI**	**Normal**	21	27.3428	4.74543	1.03554
	**Osteoporosis**	21	23.0840	5.01859	1.09515

**Table 4:** Comparison of panoramic radiographic accuracy results with DEXA results for observer I.

			**Observer (results)**			**Total**
			**Normal**	**Osteopenia**	**Osteoporosis**	
**Dexa Results**	**Normal**	Count	**13**	4	4	21
		% within Dexa Results	61.9%	19.0%	19.0%	100.0%
	**Osteopenia**	Count	3	**11**	4	18
		% within Dexa Results	16.7%	61.1%	22.2%	100.0%
	**Osteoporosis**	Count	1	4	**16**	21
		% within Dexa Results	4.8%	19.0%	76.2%	100.0%
**Total**		Count	17	19	24	60
		% within Dexa Results	28.3%	31.7%	40.0%	100.0%

**Table 5:** Average accuracy of panoramic radiographs in accordance with DEXA scan by five observers.

**PERCENTAGE**						**AVG ACCURACY**
**OBSERVERS**	**I**	**II**	**III**	**IV**	**V**	
**NORMAL**	61.9	47.6	76.2	47.6	57.1	**58.08%**
**OSTEOPENIA**	61.1	61.1	50.0	77.8	66.7	**63.34%**
**OSTEOPOROSIS**	76.2	71.4	47.6	57.1	71.4	**64.74%**
